# Rapid detection of potyviruses from crude plant extracts

**DOI:** 10.1016/j.ab.2018.01.019

**Published:** 2018-04-01

**Authors:** Gonçalo Silva, Joshua Oyekanmi, Chukwuemeka K. Nkere, Moritz Bömer, P. Lava Kumar, Susan E. Seal

**Affiliations:** a Natural Resources Institute, University of Greenwich, Chatham Maritime, Kent, ME4 4TB, UK; b International Institute of Tropical Agriculture (IITA), Oyo Road, PMB 5320, Ibadan, Nigeria; c National Root Crops Research Institute, Km 8 Ikot Ekpene Road, PMB 7006, Umudike, Nigeria

**Keywords:** Yam, Recombinase polymerase amplification, Potyvirus, Isothermal amplification, Rapid diagnostics

## Abstract

Potyviruses (genus *Potyvirus*; family *Potyviridae*) are widely distributed and represent one of the most economically important genera of plant viruses. Therefore, their accurate detection is a key factor in developing efficient control strategies. However, this can sometimes be problematic particularly in plant species containing high amounts of polysaccharides and polyphenols such as yam (*Dioscorea* spp.). Here, we report the development of a reliable, rapid and cost-effective detection method for the two most important potyviruses infecting yam based on reverse transcription-recombinase polymerase amplification (RT-RPA).

The developed method, named ‘Direct RT-RPA’, detects each target virus directly from plant leaf extracts prepared with a simple and inexpensive extraction method avoiding laborious extraction of high-quality RNA. Direct RT-RPA enables the detection of virus-positive samples in under 30 min at a single low operation temperature (37 °C) without the need for any expensive instrumentation.

The Direct RT-RPA tests constitute robust, accurate, sensitive and quick methods for detection of potyviruses from recalcitrant plant species. The minimal sample preparation requirements and the possibility of storing RPA reagents without cold chain storage, allow Direct RT-RPA to be adopted in minimally equipped laboratories and with potential use in plant clinic laboratories and seed certification facilities worldwide.

## Introduction

Plant pests and pathogens have an important role in global food crops causing significant economic losses in the agricultural industry and threatening food security [[1], [2], [3]]. Yam (*Dioscorea* spp.) is one of the most important staple food crops worldwide and plays a major role in food security and income generation for more than 60 million people in West Africa, with this region contributing over 95% of the world's total yam production [4,5]. Yams are generally propagated vegetatively through their tubers, which facilitates the spread and accumulation of pathogens, particularly viruses [6]. To date, several virus species belonging to different genera (*Potyvirus, Badnavirus, Cucumovirus, Aureusvirus, Potexvirus, Macluravirus,* and *Carlavirus*) [[7], [8], [9], [10], [11], [12]] have been reported and characterized in yams. These viral infections restrict the international exchange of yam germplasm and have a significant impact on tuber yields and quality. For example, reports from the Ivory Coast [13] and western Nigeria [14] have described average annual yield losses of 30–50% due to virus infections. Additional constraints to increase yam production and productivity are the unavailability and associated high costs of high-quality virus-free (termed ‘clean’) seed yams and the absence of a formal seed yam certification system [5,14,15].

Infections by potyviruses (genus *Potyvirus*; family *Potyviridae*) cause the most economically important diseases of yams and are widespread across the numerous yam growing regions worldwide [10,16,17]. The best described potyvirus infecting yam is *Yam mosaic virus* (YMV), known to infect several species of yam, particularly the most widely cultivated *D. rotundata*, *D. cayenensis* and *D. alata*, while the second most described yam potyvirus, *Yam mild mosaic virus* (YMMV) is more commonly found on *D. alata* [18].

Historic data suggest a strong influence of human activity on the dissemination of viruses through trade and transportation of infected plant material [1,[19], [20], [21]]. Applying full phytosanitary surveillance in plant quarantine and certification facilities is unrealistic due to high costs associated with increasing inspection rates [3]. Therefore, there is an urgent need to develop improved detection methods for yam viruses to help make timely decisions on the health status of yam planting material. Several serological and PCR-based methods have been developed and applied for the detection of YMV and YMMV [18,22,23]. Some considerations must be taken into account when choosing the detection method, such as sensitivity, specificity, cost and time to obtain results [24]. Although PCR-based assays are often preferred for their sensitivity and specificity [25], they require specific technical expertise and sophisticated equipment. In addition, PCR-based methods usually require the extraction of high-quality DNA/RNA from the sample material, which is time-consuming, generally involves hazardous chemicals and cannot be done in the field.

An isothermal amplification method called recombinase polymerase amplification (RPA) [26], overcomes the disadvantages of PCR-based assays as it reduces the need for expensive apparatus to control reaction temperature as well as providing rapid and reliable results with sensitivity and specificity comparable to conventional PCR assays [27,28]. RPA has been successfully used in the detection of several animal [[29], [30], [31]], human [32,33] and plant [[34], [35], [36]] pathogens. Recently, we developed a sensitive and robust reverse transcription-recombinase polymerase amplification (RT-RPA) assay for the specific detection of YMV [37]. To develop further this promising diagnostic method and bring it closer to a format suitable for on-site detection of the two most important yam potyviruses, the time-consuming RNA purification step needs to be removed. In this study, we report a RT-RPA method for the detection of YMV and YMMV directly from the crude extract of infected plant material using a simple and inexpensive extraction method. Yam and potyviruses form an excellent combination as a general working model of wide applicability to other plant virus systems as: (1) potyviruses comprise the largest genus of plant RNA viruses causing significant losses in different crops worldwide and (2) yams represent particularly recalcitrant leaf tissue that contain high levels of PCR-inhibitory compounds such as polyphenols and polysaccharides, and hence the technique should be suitable for application to a diverse range of plant species.

The method developed in this study, termed ‘Direct RT-RPA’, thus has the potential to be adapted to any recalcitrant plant species and be used to obtain rapid responses in certification laboratories, reducing costs by minimising quarantine time. In addition, this method will specifically strengthen current efforts in West Africa to multiply and deliver ‘clean’ certified yam planting material to smallholder farmers and thereby improve food and income security.

## Materials and methods

### Plant material

Infected and potyvirus-free (‘uninfected’) yam tubers from *D. alata* and *D. rotundata* (Table 2) were imported from the International Institute of Tropical Agriculture (IITA, Ibadan, Nigeria) and were grown in a quarantine aphid-proof glasshouse at the Natural Resources Institute (NRI, UK) as described by Mumford and Seal [18]. Individual leaf samples were collected in small polythene bags (10 × 15 cm) and used immediately.

**Table 1:** RPA primers and probes sequences used in this study.

Name	Sequence (5′ – 3′)	Product size (bp)	Source
YMV RPA 3F	CAAATTTATCCGGRATGTGGACRATGATGGAC	121	Silva et al., 2015
YMV RPA 3R	GCGTCACTRAAATGCATCATTATYTGACGAAA		
Probe YMV exo 3F/3R	TGTGGGTTTGGCATTTTCTATGATCGGTT(F)C(Z)A(Q)GGATATTCCACTT-Spacer C3		
YMMV RPA F2	ACACATGCAAATGAARGCAGCAGCTYTRCG	264	This study
YMMV RPA R2	TGAAYCACCAGTAGAGTGAACATAGTAYTTA		
Probe YMMV exo F2/R2	TGCACTCNCTYCTTGGAGTGCGYAAYATC(F)A(Z)A(Q)ATTTATATAAGTAA-Spacer C3		

(F) = FAM-dT: thymidine nucleotide carrying FAM fluorophore; (Z) = tetrahydrofuran residue; (Q) = BHQ1-dT: thymidine nucleotide carrying Blackhole Quencher-1.

**Table 2:** Comparison of RT-RPA and RT-PCR for detection of YMV and YMMV from purified RNAs and crude plant extracts from yam samples. Detection of the yam actin gene by RT-PCR was used as an internal control.

Sample accession number	YMV				YMMV			
	RT-RPA (min)		RT-PCR		RT-RPA (min)		RT-PCR	
	RNA	Crude extract	RNA	Crude extract	RNA	Crude extract	RNA	Crude extract
TDa 95/310	−	−	−	−	−	−	−	−
TDa 98/159	−	−	−	−	10.32	11.38	+	−
TDa 98/01166	−	−	−	−	6.57	10.69	+	+
TDa 99/00240	−	−	−	−	−	−	−	−
TDa 00/00005	−	−	−	−	11.90	13.37	+	−
TDa 00/00194	−	−	−	−	12.18	12.79	+	−
TDr 89/02475	−	−	−	−	−	−	−	−
TDr 07/00033	3.10	5.05	+	−	−	−	−	−
TDr 99/02674	5.46	6.12	+	−	−	−	−	−
TDr 02/00515	4.37	5.17	+	+	−	−	−	−
TDr 95/19177	5.05	4.21	+	−	−	−	−	−
TDr 00/00515	5.17	4.60	+	+	−	−	−	−
TDr 00/00168	4.79	5.05	+	−	−	−	−	−
TDr 00/00362	5.42	7.00	+	−	−	−	−	−
TDr 89/02665	5.09	25.34	+	−	−	−	−	−
TDr 96/00604	6.12	10.97	+	−	−	−	−	−
TDr 07/00873	3.50	8.43	+	+	−	−	−	−
TDr 03/00196	3.36	5.63	+	−	−	−	−	−
Ogoja (TDr)	−	−	−	−	−	−	−	−
Hembakwase (TDr)	4.74	7.21	+	+	−	−	−	−
Idu-Ekpeye (TDr)	7.67	7.01	+	+	−	−	−	−
Pepa (TDr)	9.86	23.17	+	−	−	−	−	−
Ogini (TDr)	−	−	−	−	−	−	−	−
Pouna (TDr)	7.37	5.59	+	−	−	−	−	−
Nwopoko (TDr)	5.06	7.23	+	+	−	−	−	−
Aloshi (TDr)	−	−	−	−	−	−	−	−
Obioturugu (TDr)	−	−	−	−	−	−	−	−
Adaka (TDr)	4.74	24.15	+	+	−	−	−	−
Amola (TDr)	−	−	−	−	−	−	−	−
Makakusa (TDr)	−	−	−	−	−	−	−	−

+: Positive result; −: negative result; TDa: *Dioscorea alata* accession; TDr: *Dioscorea rotundata* accession.

### RNA extraction and crude sample preparation

**Fig. 1:**
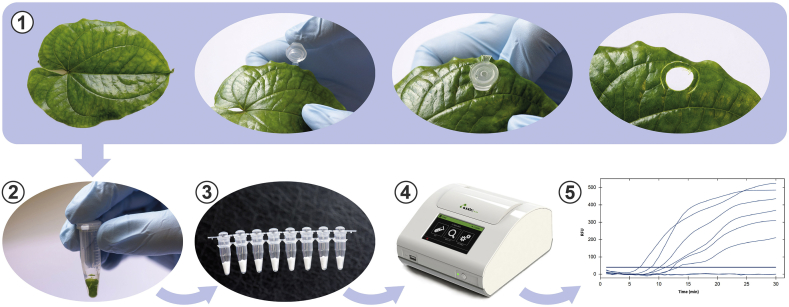
Direct RT-RPA detection method workflow showing sample processing and assay setup steps. 1 - punch leaf with lid of 1.5 mL tubes; 2 - immerse leaf disk in alkaline-PEG buffer and incubate at room temperature for <5 min; 3 - resuspend lyophilised pellet; 4 - read fluorescence; 5 - analyse results: an increase in fluorescence above threshold indicates a positive reaction.

Extraction of total RNA from leaf tissue (∼100 mg) was performed using RNeasy Plant Mini Kit (Qiagen, Manchester, UK) according to the manufacturer's instructions. The final pellet was resuspended in 50 μL nuclease-free water and stored at −80 °C prior to testing. Total RNA was quantified using a Nanodrop 2000 spectrophotometer (Thermo Scientific, Loughborough, UK). For the detection of YMV and YMMV from crude plant extracts, a modified alkaline polyethylene glycol (PEG) extraction method [38] was used, as described by Hwang et al. [39]. The same leaf samples used for RNA extraction were used to prepare crude extracts: leaf disks (∼13 mg) obtained with the lids of 1.5 mL tubes were immersed in 300 μL of freshly prepared alkaline-PEG buffer (6% PEG 200 (Sigma-Aldrich, Gillingham, UK) with 20 mM NaOH). Tubes were vortexed briefly and then incubated at room temperature for <5 min (Fig. 1) making sure leaf disks were soaked in the buffer. Plant extracts were tested immediately or kept on ice until further use.

### Conventional RT-PCR

The presence of YMV and YMMV was confirmed by RT-PCR using the primer pairs YMV-F/-R and YMMV-F/-R [18], which amplify a 586 bp and a 249 bp region comprising the coat protein (CP) gene and the 3′ UTR region of the YMV and YMMV genomes, respectively. An assay for detection of the yam actin gene was used as an internal control as described by Silva et al. [37]. RT-PCR amplifications were set up in 20 μL reactions containing either 40 ng RNA or 2 μL of crude extract, 0.2 μM of each primer, 0.25 mM of each dNTP, 2.5 U AMV Reverse Transcriptase (Promega, Southampton, UK), 1 U DreamTaq DNA polymerase and 1x DreamTaq Green buffer (Thermo Scientific, Loughborough, UK) containing 2 mM MgCl_2_. The following cycle conditions were used: 50 °C for 10 min for reverse transcription, 95 °C for 4.5 min, followed by 30 cycles of 95 °C for 30 s, 55 °C for 1 min, 72 °C for 1 min and one final extension of 72 °C for 5 min. Amplification products were analysed by agarose gel electrophoresis using 1.5% (w/v) agarose gels containing 1x RedSafe nucleic acid stain (iNtRON Biotechnology, Seongnam, South Korea) in 0.5x Tris-Boric acid-EDTA (TBE) buffer.

### RT-RPA

RPA primers and probe for YMV were as previously described [37]. New RPA primers and probe were designed to the YMMV coat protein gene by performing a multiple sequence alignment of YMMV nucleotide sequences available in the National Centre for Biotechnology Information (NCBI) GenBank database. Sequences of primers and probes used for RPA assays are shown in Table 1. All the primers described in this study were synthesised by Sigma-Aldrich (Gillingham, UK), and TwistAmp exo probes were synthesised by Eurogentec S.A (Seraing, Belgium).

RPA was performed using the materials and protocols provided with the TwistAmp exo-RT kit, which already contains a reverse transcriptase in the dried enzyme pellet (TwistDx, Cambridge, UK). RT-RPA reactions were performed in 10 μL reaction volume using the enzyme pellets of the TwistAmp exo-RT kit, 6U of RiboLock RNase Inhibitor (Thermo Scientific, Loughborough, UK), 420 nM of both RPA primers, 120 nM of RPA exo probe, 14 mM magnesium acetate and TwistAmp rehydration buffer. A master mixture containing all reagents except for the magnesium acetate and template was prepared and used to rehydrate the dried enzyme pellets. This solution was then aliquoted into 0.2 mL PCR tubes (8.5 μL/tube) and 1 μL (corresponding to 20 ng) of purified RNA (for ‘RT-RPA’) or crude extract (for ‘Direct RT-RPA’) was added to the reaction mixture (Fig. 1). To initiate the reaction, magnesium acetate was pipetted into the cap of each tube. Subsequently, tubes were recapped and centrifuged briefly. Fluorescence measurements in the FAM channel were performed in a real-time PCR instrument (CFX96 Touch™ Real-Time PCR Detection System, Bio-Rad, Hemel Hempstead, UK) at 37 °C every 1 min for 30 min. Analysis of fluorescence intensity over time on baseline subtracted data and threshold calculations were done using CFX Manager™ software. Samples that produced a fluorescent amplification curve above the threshold were considered positive. Negative amplifications did not show an increase in fluorescence signal.

The sensitivity of the ‘Direct RT-RPA’ was evaluated using serial dilutions of the crude plant extracts. The initial extract, obtained from a YMV or YMMV-infected plant in a 1:20 (*w/v*) dilution was considered to be a 10^0^-fold dilution and then further diluted in 10-fold series, unless otherwise stated, with crude extracts from the uninfected plants. Each dilution was tested in 5 replicates.

## Results

### Direct RT-RPA assays for YMV and YMMV detection

**Fig. 2:**
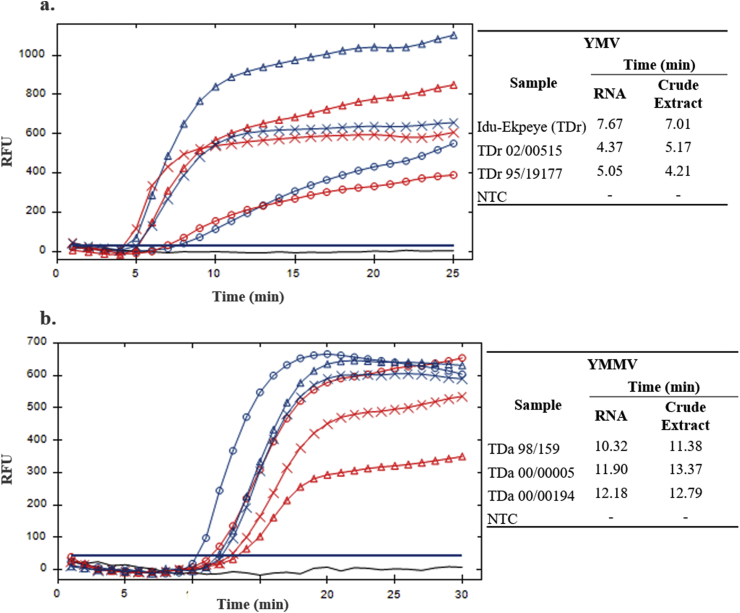
RT-RPA, with purified RNAs as template and Direct RT-RPA, with crude plant extracts as template for detection of YMV (a.) and YMMV (b.). Blue lines are obtained from purified RNAs and red lines obtained from crude plant extracts. Circle corresponds to Idu-Ekpeye (TDr) and TDa 98/159 samples; Triangle corresponds to TDr 02/00515 and TDa 00/00005 samples; Cross corresponds to TDr 95/19177 and TDa 00/00194 samples. NTC refers to the non-template control. Fluorescence intensities were plotted against time in minutes. The solid bar corresponds to the threshold line (graph generated by CFX ManagerTM software). Tables on the right side of the amplification plots show the threshold times (min) of each reaction. (For interpretation of the references to colour in this figure legend, the reader is referred to the Web version of this article.)

Purified total RNAs and crude plant extracts from yam samples were used for the detection of both YMV and YMMV by RT-RPA using a single incubation temperature of 37 °C for a maximum of 30 min analysis period. Fig. 2 shows a typical result obtained by RT-RPA, with purified RNAs as template and Direct RT-RPA, with crude plant extracts as template. YMV was found only in *D. rotundata* accessions (TDr) and YMMV only in *D. alata* (TDa) samples. No double infections were found in any of the samples analysed.

Specificity of the assay was confirmed by cross reaction assays, i.e. testing YMV and YMMV primers and probes in YMMV- and YMV-positive samples, respectively. No cross reactions occurred for any of the samples (Table 2), indicating a high specificity of the primers and probes used for each specific target. Positive amplification signals above threshold were achieved within 15 min.

Furthermore, results for Direct RT-RPA agreed with those for RT-RPA for all samples analysed and similar amplification times were obtained for both assays for each target (Fig. 2 and Table 2).

### Comparison of direct RT-RPA and conventional RT-PCR

The same crude plant extracts and purified RNAs used for RT-RPA were analysed by RT-PCR (Table 2). All samples that were negative by RT-RPA (with either purified RNA or crude plant extract) were also negative by RT-PCR. Negative results for YMV and YMMV being due to a lack of viral RNA were confirmed by amplifying the actin housekeeping gene by RT-PCR as internal control (results not shown). Results showed that when using RNA as template for YMV and YMMV detection similar results were obtained with RT-RPA and RT-PCR. However, when analysing crude plant extracts the RT-PCR was unable to detect the virus infection in 13 samples (TDa 98/159; TDa 00/00005; TDa 00/00194; TDr 07/00033; TDr 99/02674; TDr 95/19177; TDr 00/00168; TDr 00/00362; TDr 89/02665; TDr 96/00604; TDr 03/00196; Pepa; Pouna), resulting in false-negative results.

Using the detection time for comparison between both techniques, the Direct RT-RPA required less time to detect the target template than the standard RT-PCR. All Direct RT-RPA reactions were achieved in under 15 min, except for 3 samples (TDr 89/02665; Pepa; Adaka) compared to > 150 min required for RT-PCR.

### Sensitivity of the direct RT-RPA assay

Serial dilutions of crude plant extracts obtained from either YMV- or YMMV-infected yam plants were tested by Direct RT-RPA for its detection limit (Table 3). Five reactions were performed for each dilution. YMV was detected consistently down to the 1×10^−3^ dilution. For YMMV, on the other hand, only the original crude plant extract and 10^−1^ dilutions were detected consistently. No results were obtained at dilutions lower than 2×10^−3^.

**Table 3:** Limit of detection of YMV and YMMV by Direct RT-RPA assay. Crude plant extracts obtained from either YMV- or YMMV-infected yam plants were serially diluted with crude plant extracts from uninfected yam plants. The number of positive test results for both viruses is presented in relation to the total number of tests performed at each dilution.

Dilution	Limit of detection (positive replicates/total tested)	
	YMV	YMMV
10^0^	5/5	5/5
1×10^−1^	5/5	5/5
1×10^−2^	5/5	2/5
2×10^−3^	5/5	0/5
1×10^−3^	5/5	0/5
5×10^−4^	4/5	0/5
2×10^−4^	2/5	0/5
1×10^−4^	1/5	0/5

## Discussion

The lack of ‘clean’ seed yams is a major constraint to improve yam productivity in West Africa. In fact, the accumulation of viruses in the yam vegetatively propagated germplasm has led to an endemic situation in the West African ‘yam belt’, a region that extends from Western Cameroon to Nigeria, Benin, Togo, Ghana, and Côte d’Ivoire. Njukeng et al. [17] further concluded that another main factor contributing to the high incidence and distribution of viruses infecting yam was the lack of sensitive and field-based diagnostic tools. Therefore, the development of robust and low-cost diagnostic methods is critical to assist the production and certification of disease-free seed yams.

Recombinase polymerase amplification [26] is an ideal method for point-of-care diagnostics and a good alternative to PCR because it is significantly faster and does not require expensive laboratory equipment [27,40]. Silva et al. [37] presented the first use of RT-RPA for the detection of yam viruses from purified RNA. In the present study, this assay was adapted to detect both main potyviruses, YMV and YMMV, using crude plant extracts from infected yam material, a method termed ‘Direct RT-RPA’. With this method, there is no need for detailed nucleic acid extraction protocols, and both viruses are detected accurately from plant extracts. The 20 mM NaOH present in the alkaline-PEG buffer increases the pH of the solution needed for an effective lysis of plant cells. High pH solutions can degrade RNA, however the buffer also includes PEG which plays a role in the neutralization of the solution after cell lysis and thereby reducing the inhibition from the high pH.

In contrast to Direct RT-RPA, RT-PCR is susceptible to inhibitory compounds present in crude yam plant extracts leading to false-negative results detrimental for certification of disease-free seed yams.

The Direct RT-RPA showed high specificity for each virus, but greater dilutions could be made for YMV compared to YMMV infected leaf material. Substances present in the sample matrix can interfere with the enzymatic nucleic acid amplification. Previous studies [[41], [42], [43]] have reported that the presence of background DNA or specific concentration of ions in the sample could have a negative influence on the RPA sensitivity. In our study, the lower limit of detection obtained for YMMV could be explained by the higher levels of polysaccharides contained in extracts obtained from *D. alata* compared to *D. rotundata* leaves [18]. We tried to improve the YMMV Direct RT-RPA sensitivity by including a mixing step after approximately 4 min of incubation, as suggested by Lillis et al. [40] and TwistDx. We compared reactions with and without a mixing step in 5 replicates but our results suggested that the mixing step had an opposite effect and sensitivity was further compromised (data not shown). Nevertheless, the detection limit of the Direct RT-RPA obtained for both viruses is better or equivalent to that of ELISA obtained by Eni et al. [23] or the RT-RPA assay developed by Mekuria et al. [34], who reported that *Little cherry virus 2* (LChV2) could be successfully detected in a 1×10^−2^ dilution of crude leaf extracts.

In this study, the reaction temperature was set and fluorescence signal were measured using a laboratory real-time PCR instrument. The Direct RT-RPA could amplify the target RNA at a relatively low and isothermal incubation temperature of 37 °C, which is near ambient temperature experienced in West Africa. However, and as reported by others [27,28,44], RPA tolerates temperature fluctuations between 25 °C and 42 °C without the performance of the reaction being compromised. This means that accurate detection can be achieved using simple, battery-powered portable instruments reducing the cost of the assay [33,45]. Other factors contributing to the reduction of cost of the Direct RT-RPA include the use of crude plant extracts as templates and the time needed to obtain results; Direct RT-RPA can be completed in less than 30 min.

A major advantage of the Direct RT-RPA is that samples can be prepared in a few minutes without the need of hazardous chemicals and reducing the user-dependent steps in the protocol. The combination of RT-RPA with minimal sample preparation requirements for plant virus detection makes Direct RT-RPA suitable for integration into automated sample-to-answer microfluidic platforms [28], similar the ones developed for the detection of methicillin-resistant *Staphylococcus aureus* [46] and *Yellow fever virus* [47], which will facilitate the adaptation of this method to a format suitable to on-site applications, in particular in resource limited settings. This is further supported by recent reports demonstrating the stability of RPA reagents at elevated temperatures up to 45 °C [27,40], avoiding dependence on cold chain storage during transportation.

In conclusion, a rapid and robust Direct RT-RPA method for the detection of the two main potyviruses infecting yam has been developed (Fig. 1). With minimal sample preparation requirements, the Direct RT-RPA showed high tolerance to plant inhibitors (polysaccharides and polyphenols) and good specificity and sensitivity. Overall, this method has demonstrated to be a promising alternative to the conventional RT-PCR in current general use and has the potential to be used in certification facilities to assist in the rapid selection of virus-free yam planting material or any other recalcitrant plant species.

## Author contributions

GS and SES conceived and designed the experiments; GS, JO and CKN performed the experiments; GS, JO, CKN, MB and PLK analysed the data; PLK contributed materials; GS drafted the manuscript; All authors edited, read and approved the final manuscript.

## Conflicts of interest

The authors declare no conflict of interest.

## Abbreviations

**The following abbreviations are used in this manuscript**
bp: base pairs
CP: coat protein
NaOH: sodium hydroxide
NCBI: National Centre for Biotechnology Information
PCR: polymerase chain reaction
PEG: polyethylene glycol
RPA: recombinase polymerase amplification
RT-PCR: reverse transcription-polymerase chain reaction
RT-RPA: reverse transcription-recombinase polymerase amplification
TDa: *Dioscorea alata* accession
TDr: *Dioscorea rotundata* accession
YMV: *Yam mosaic virus*
YMMV: *Yam mild mosaic virus*
